# Structure-Dependent Properties of Silver-Decorated 3D-Reduced Graphene Oxide Nanocomposites: Influence of Reagent Addition Sequence and Applications in Electrochemical Sensing

**DOI:** 10.1021/acsomega.4c09382

**Published:** 2025-04-17

**Authors:** Paulo Castro Cardoso da Rosa, Anna Elisa Silva, Eduardo Guilherme Cividini Neiva, José Rafael Bordin, Carolina Ferreira de Matos Jauris

**Affiliations:** † Environmental Science and Technology Center, Federal University of Pampa, Caçapava do Sul 96570000, Brazil; ‡ Department of Chemistry, Regional University of Blumenau, Campus 1, Blumenau 89030-903, Brazil; § Department of Physics, Institute of Physics and Mathematics, Federal University of Pelotas, Caixa Postal 354, Pelotas 96001-970, Brazil; ∥ Department of Chemistry, Federal University of Santa Maria, Santa Maria 97105-900, Brazil

## Abstract

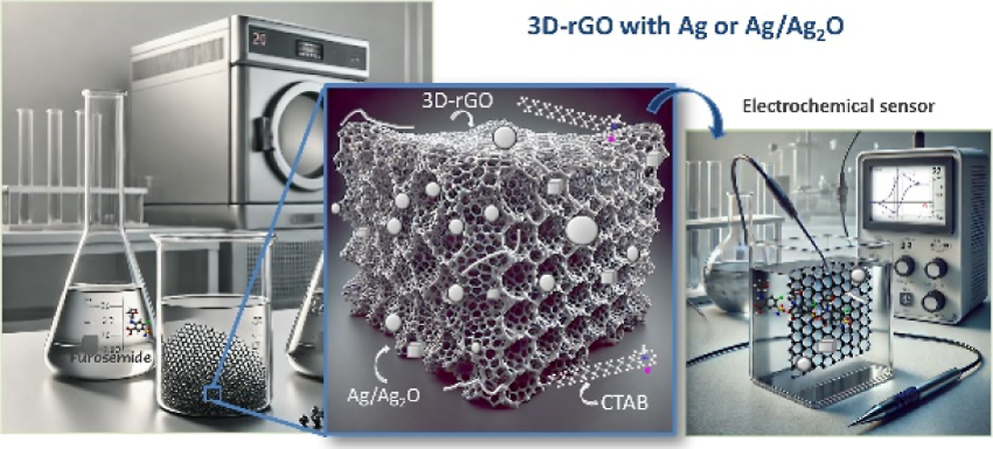

This work aimed to demonstrate how careful control of the reagent concentration and the order of their addition can be used to fine-tune the characteristics of 3D-reduced graphene oxide structures decorated with silver and how this affects the applicability of these materials as electrochemical sensors. The materials were prepared by using an environmentally friendly single-step route in an autoclave, using only water as a solvent and ascorbic acid as a reductant, while varying the order of addition of the reagents and the amount of metal precursors. The presence of cetyltrimethylammonium bromide (CTAB) surfactant leads to the formation of Ag_2_O particles in addition to pure Ag. A greater quantity of metal precursors resulted in a more compact macrostructure with smaller particles. The late addition of CTAB promoted the formation of smaller silver nanoparticles, which preferentially decorated the edges and folds of the rGO sheets. Computational calculations allowed for the elucidation of the mechanism responsible for this preferential morphology. The main advantage of the method used is its ability to synthesize simultaneously and in large quantities different materials in a fast, single-step approach. The synthesis route can influence the formation and characteristics of the silver particles, such as their composition, size, and shape. This architecture creates efficient conduction networks with maximum utilization of spaces and interfaces, acting as a conductive layer for the Ag or Ag/Ag_2_O nanoparticles that decorate the macrostructure. The macrostructures showed applicability in furosemide sensing, with LD and LQ reaching 21 ± 2 and 69 ± 8 μmol L^–1^, respectively.

## 1 Introduction

Graphene-based materials are the most prominent revolution in materials science due to their excellent properties, and it is fascinating to see how we can improve these properties and the multifunctionality of these materials. For example, 3D graphene-based materials can be obtained through the three-dimensional rearrangement of the layers of the material. Controlling the self-assembly of the individual graphene sheets can prevent its stacking, forming a cohesive and stable macroporous structure with a large surface area and properties that are not observed in their 2D counterparts. Low density, high mechanical properties, thermal stability, high electrical conductivity, and high specific capacitance characterize 3D-based graphene materials.^1,2^ Different macroporous systems are based on graphene species, such as foams, sponges, hydrogels, and aerogels.

More interestingly, we can improve these properties and even add new ones through material functionalization. The 3D networks with high specific surface area and porosity allow the growth and anchoring of inorganic nanomaterials with a high amount of charge, significantly enhancing the synergistic effect of the two components. In this sense, 3D graphene can be decorated with many molecules and nanoparticles, providing these materials significant advantages, especially concerning their excellent electrochemical and catalytic activity.^3^

Silver nanoparticles decorating graphene materials were the subject of many studies.^4−6^ Different synthesis methods are applied in these works, and each allows the production of nanoparticles with different morphologies, sizes, and shapes, providing them with different characteristics and functions. Despite the importance of reaction controls, studies addressing the effects of the reagent addition sequence and metal precursor concentration on the formation of decorated 3D-rGO-based structures remain scarce.

Another gap in the literature lies in exploring the application of this type of nanocomposite as an electrochemical sensor, particularly in understanding its performance, sensitivity, and selectivity. In this regard, a few studies about the application of tridimensional rGO-Ag-based nanocomposites have been done as electrochemical sensors. Recently, Zhang and co-workers^7^ prepared by a green method an ultrasensitive, electrochemical sensor based on Ag nanoparticle-anchored 3D rGO for rifampicin detection with a detection limit of 0.810 nmol/L. Sawangphruk et al.^8^ used 3D-Ag/graphene aerogel composites to immobilize the enzyme and prepare biosensors to determine low concentrations of the allergen sulphite. Zhao et al.^9^ prepared hybrid Ag NPs dispersed on electrochemical sensors for hydrogen peroxide.

In this work, we investigate the influence of variations in reagent addition order and precursor concentration on the structure-dependent properties and electrochemical sensing applications of silver-decorated, 3D-reduced graphene oxide nanocomposites. We use large-scale coarse-grained simulations to gain molecular-level insight into the synthesis process. The second section details the experimental procedures and simulation methodologies. In Section 3, we present and discuss our findings, showcasing how the material’s structural and thermal properties were characterized and how simulations provided a deeper understanding of its formation. The next section highlights the material’s electrochemical characteristics and their application as a sensor for the furosemide drug molecule. Finally, in the last part, we conclude with key insights and future research directions.

## 2 Experimental and Computational Details

### 2.1 One-Step Synthesis of 3D-Graphene-Based Macrostructures with Silver Particles

The synthesis started from the chemical graphite oxidation (20 μm graphite powder, purchased from Sigma-Aldrich), followed by exfoliation of the graphite oxide through an ultrasound bath to obtain graphene oxide (GO).^10^ The 3D-graphene macrostructures with silver particles were prepared by using different routes, changing the concentration and order of the addition of the reagent.

The sample control was obtained as described in our previously published works:^11−13^ 25 mL of the GO dispersion (2.2 mg mL^–1^) was mixed with 90 mg of the reducing agent ascorbic acid (Synth, 99% purity) and 25 mL of distilled water. Next, the beakers containing all of the mixtures were inserted in an autoclave (Stermax Digital Super Top) for 90 min at 120 °C and 3.5 bar of pressure. After that period, the equipment remained off and was reopened after cooling. Finally, the obtained materials were removed, washed, filtered with distilled water, and placed in an oven at 60 °C until dry. This sample will be denominated as *control.*

To evaluate the effect of the metal concentration, two different materials were synthesized containing two different proportions of metal precursor (AgNO_3_—Sigma-Aldrich). For this purpose, 25 mL of the GO dispersion (2.2 mg mL^–1^) and 90 mg of the ascorbic acid were mixed with 1.7 or 5.1 mg of AgNO_3_ previously dispersed in 25 mL of distilled water (10 or 30 mmol L^–1^). The choice of these ratios was based on the proportions used in different studies from the literature.^14−16^ Finally, the samples were kept in an autoclave, washed, and dried under the same conditions used in the sample control. These samples will be denominated as *10Ag and 30Ag.*

Two different materials were synthesized to evaluate the effect of the cationic surfactant hexadecyltrimethylammonium bromide (CTAB) presence. For this purpose, 25 mL of the GO dispersion (2.2 mg mL^–1^), 90 mg of the reducing agent ascorbic acid, and 5 mL of a 1 mg mL^–1^ CTAB solution (ACROS, 99% purity) were mixed with 1.7 or 5.1 mg of AgNO_3_ previously dispersed in 20 mL of distilled water (10 or 30 mmol L^–1^). The samples were kept in an autoclave, washed, and dried in the same conditions used in the sample previously described. These samples will be denoted as *CTAB + 10Ag* and *CTAB + 30Ag.*

Two samples were prepared to evaluate the reagent order addition effect; the proportions were the same as those used in the syntheses above, but with an order inversion between the addition of the metallic precursor and the surfactant, where the precursor was first added and soon after the CTAB. These samples will be denominated as *10Ag + CTAB and 30Ag + CTAB.*

To achieve better sample homogeneity and initially improve the distribution of silver ions on the GO surface, the mixture was subjected to an ultrasonic bath for 5 min at each step of the addition process. The addition of CTAB was specifically intended to control the particle size and prevent the formation of aggregates. The amounts of the reactants and their addition order used in the material preparation are summarized in Table 1.

**Table 1:** Amounts of the Reactants and Their Addition Sequence Were Used to Prepare the Different Macrostructures

addition sequence/reagent				
addition sequence	1st	2nd	3rd	4th
reagent	GO dispersion (mL)	ascorbic acid (mg)	CTAB solution (mL)	AgNO_3_ (mg)
control	25	90	0	0
10Ag	25	90	0	1.7
30Ag	25	90	0	5.1
CTAB + 10Ag	25	90	5	1.7
CTAB + 30Ag	25	90	5	5.1

addition sequence/reagent				
addition sequence	1st	2nd	3rd	4th
reagent	GO dispersion (mL)	ascorbic acid (mg)	AgNO_3_ (mg)	CTAB solution (mL)
10Ag + CTAB	25	90	1.7	5
30Ag + CTAB	25	90	5.1	5

### 2.2 Characterizations

The X-ray diffractograms were obtained in Rigaku Brand equipment and ULTIMA IV model, with Bragg–Brentano geometry and Cu Kα wavelength (λ = 0.15418 nm). Measurement conditions: 40 kV/20 mA power, a step of 0.05°, and an integration time of 1 s. Thermogravimetric analysis (TGA) was performed on a NETZSCH TG 209 F1 equipment under a nitrogen atmosphere, starting from room temperature to 900 °C, at a heating rate of 10 °C min^–1^. These characterizations were performed in Thermal Analysis. The scanning electron microscopy (SEM) images of the surfaces of the three-dimensional graphene samples were obtained using a Tescan microscope model MIRA3 FEG-SEM, using an In-beam detector. The voltage of the source used was 10 kV. The Raman spectra were obtained using a Renishaw Raman Imaging Microscope System 3000. The excitation line used was He-Ne-633 nm operating at 32 mW power using a 50× objective.

### 2.3 Computational Model and Details

This computational approach aimed to understand the molecular details involved in the material decoration of silver and the role of the surfactant in the process. Due to the complexity of the solution, comprising the graphene macrostructure, surfactant, silver ions, and counterions to neutralize the system, we employed a coarse-grained (CG) method based on the implicit solvent Dry Martini force field.^10^ In this approach, the same bead types from the standard Martini force field are used. However, the interaction strengths are modified to account for the implicit consideration of water.

The CG model allowed us to simulate systems with the same concentrations as in the experiments. CTAB was modeled following the Illa-Tuset approach et al.^17^ In this model, the surfactant’s hydrophobic tail beads are represented as C1Martini beads, while the polar head is modeled as a Q0 bead. The beads in each surfactant molecule are connected by harmonic springs, and a bending spring was used to capture the molecule’s rigidity. To model the oxidized graphene sheets, we followed the method of Titov et al.,^18^ where carbon-like atoms are modeled as C1Martini particles with a 60% reduction in the Lennard-Jones (LJ) parameters. Oxidized sites, when present, are randomly selected and are represented as P1 monomers. For simplicity, both graphene and graphene oxide beads were not integrated over time. Silver cations were modeled as Qd beads with a charge of +e, while the counterions were modeled as Qa beads, representing a hydrated anion.

Langevin dynamics simulations were performed using the package ESPResSo.^19^ Periodic boundary conditions were applied in all directions, and the equations of motion were integrated using the Velocity-Verlet scheme with a 22.5 fs time step. The temperature was fixed at 300 K. The simulation box has a size of 100.0 nm × 100.0 nm × 50.0 nm in the *x*, *y,* and *z* directions, respectively. As proposed by Wang and Larson,^20^ the Dry Martini package may require an artificially high dielectric constant for water to depict the formation and rupture of surfactant micelles properly. In this way, the electrostatic interactions of our implicit water system have ε_r_ = 150 and were handled using the P3M algorithm, as implemented in the ESPResSo package, where the cutoff distance and Ewald Summation parameters are evaluated and optimized for each simulation. For the Lennard-Jones interaction, a 1.2 nm cutoff was used.

The system consists of two graphene or graphene oxide sheets placed at the simulation box center in the *z*-direction. The separation is 5.0 nm; therefore, we can check for differences between the aggregation at the sheet center and border. Initially, distinct concentrations (0, 10, or 30 mmol L^–1^, as in the experiments) of silver, surfactant, and counterions are added randomly. Then, we allowed the system to evolve for 22.5 ns for the equilibration process. It is followed by a 2.25 μs production stage. We track the total and kinetic energies and the pressure along all simulations to ensure that the system is well equilibrated.

### 2.4 Electrochemical Sensors

The electrochemical measurements were realized in a PalmSens potentiostat using a three-electrode system with carbon paste electrodes (CPEs) as the working electrode (WE), a Pt wire as an auxiliary electrode (AE), and Ag/AgCl (NaCl saturated) as the reference electrode (RE) in a 0.1 mol L^–1^ KOH aqueous solution. Before preparing the CPE, the 3D samples were powdered in a mortar with a pestle to ensure better homogeneity. Next, the CPE was prepared by mixing the macrostructures, nujol (Mantecorp), and graphite (Fisher Chemical) in 20, 20, and 60% of mass proportion for 20 min in a mortar with a pestle. Then, the carbon pastes were packed into the cavity of a plastic tube (depth of 1 mm and internal diameter of 1.4 mm) using a Cu wire as an electric contact, which was previously planned, polished, and cleaned. Next, glossy paper was used to smooth the paste surface. A non-modified CPE was also prepared using 20% nujol and 80% graphite. The cyclic voltammograms were recorded in the potential range of 0.00 to 0.85 V at 50 mV s^–1^. The sensing of furosemide (Fagron) through chronoamperometry was carried out with a magnetic bar of 1 cm in length under magnetic stirring (500 rpm). The chronoamperograms were recorded after the voltammetric pretreatment to reach current stability. Furosemide was chosen as the analyte due to its significance as an important molecule of environmental and biological interest.

## 3 Results and Discussion

### 3.1 Morphology

Photographic images of the freshly prepared macrostructures are shown in Figure S1 in the Supporting Information. The initial characteristics of the macrostructures demonstrate that the synthesized materials have a cylindrical shape due to the synthesis being carried out in a beaker cup. In addition, the control, 10Ag, and 30Ag samples are self-supporting, cohesive, and more compact, preventing their fragmentation and carrying by water. The CTAB + 10Ag, CTAB + 30Ag, 10Ag + CTAB, and 30Ag + CTAB materials showed some unpacking of the structure and, consequently, a decrease in their cohesion, which is probably related to the adhesion of CTAB to the sheets of the GO, hindering a chemical connection between the sheets during the reduction process.^21,22^ Materials with similar characteristics were prepared and described by Han et al.^23^

**Figure 1:**
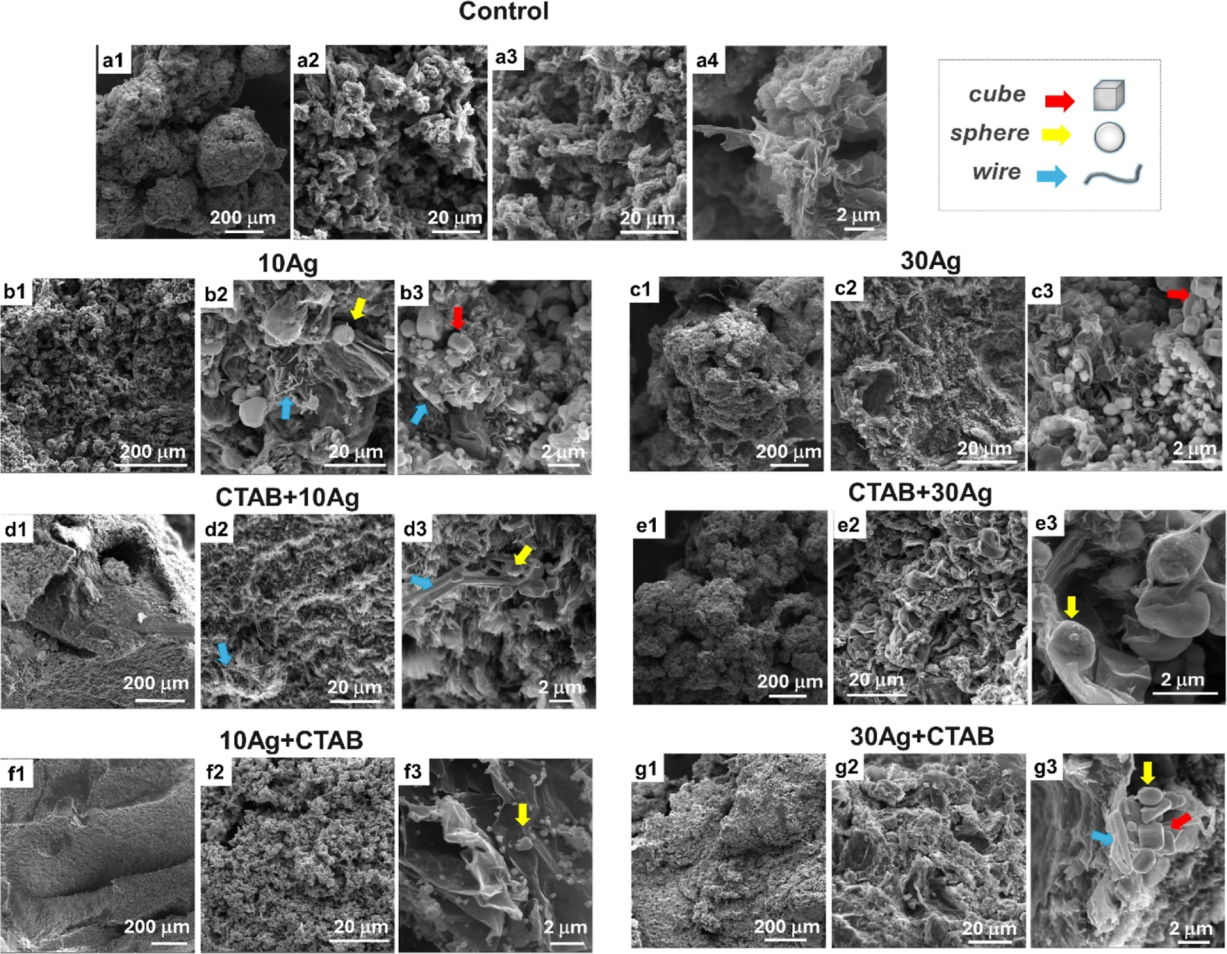
SEM images of samples: Control (a1–a4), 10Ag (b1–b3), 30Ag (c1–c3), CTAB + 10Ag (d1–d3), CTAB + 30Ag (e1–e3), 10Ag + CTAB (f1–f3), and 30Ag + CTAB (g1–g3). The yellow arrows in the images indicate spherical particles, the red arrows indicate cubes, and the blue arrows indicate silver wires.

The structural organization, pores, and particle size were evaluated by using scanning electron microscopy (SEM). The results shown in Figure 1a1–a4,b1–b3,c1–c3,d1–d3,e1–e3,f1–f3,g1–g3 represent, respectively, the samples, Control, 10Ag, 30Ag, CTAB + 10Ag, CTAB + 30Ag, 10Ag + CTAB, and 30Ag + CTAB.

For the control sample, without adding the silver precursor agent, in Figure 1a1–a4, we observe a structure with disorderly interconnected sheets, forming a three-dimensional structure. This behavior can be associated with the high pressure that “crushes” the GO sheets and generates an approximation between them, allowing their union and, consequently, the formation of the macrostructure. At the same time, the temperature (120 °C) causes the water molecules (liquid and vapor) to remain between the GO, giving rise to the pores, thus forming a porous graphene oxide macrostructure, which is simultaneously reduced by the ascorbic acid.

For the material 10Ag (Figure 1b1–b3), with the addition of 10 mM AgNO_3_, and material 30Ag (c1–c3), with the addition of 30 mM AgNO_3_, it is possible to note that the GO sheets are decorated with particles, which have a size ranging from nanometers to micrometers and different formats, such as wire spheres and cubes. To provide greater clarity, Figure S2 presents secondary electron and backscattered electron images of the same region of the CTAB + 10Ag sample. These images effectively illustrate the distribution and morphology of the particles. The histogram of the nanoparticle size is shown in Figure S3. It is observed that as the precursor concentration increases, there is a decrease in the nanoparticle size and an improvement in their homogeneity. This phenomenon can be attributed to the supersaturation of the medium, which creates more favorable conditions for nucleus formation. As nucleation occurs rapidly, a greater number of nuclei are generated simultaneously. Consequently, the amount of material available for the growth of each nucleus is reduced, leading to smaller nanoparticles. The micrometric size can be justified by the absence of a stabilizer in this material since the coalescence of the silver nanoparticles occurs to reduce their surface energy.^24,25^ The addition of silver ions in the preparation of the macrostructures and the formation of the macrostructure allowed the decoration of reduced graphene oxide (rGO) by silver particles in a single synthesis step. In addition, there is an increase in compaction between the rGO sheets when silver is added compared with the control material. Moreover, the compaction increases when the amount of silver increases, indicating that the silver particles possibly link to the rGO sheets, forming a more compact macrostructure. A similar behavior was observed by Tang et al.,^26^ where metal nanoparticles played a key role in creating a 3D graphene network so that metal nanoparticles anchored in the GO sheet could act as active sites for joining and assembling with another GO sheet. It is noted that the particles are not only over the rGO sheet but are also inside the 3D structure, indicating that for these samples, the sheets also act as a stabilizer, preventing the growth of part of the nanoparticles.

Figure 1d1–d3,e1–e3 shows the SEM images for the materials CTAB + 10Ag and CTAB + 30Ag, respectively, where it is possible to observe the formation of silver particles that decorate the rGO sheets. These images revealed exciting details about the 10Ag + CTAB material, as seen in Figure 1f1–f3. First, this graphene-based three-dimensional material was homogeneously decorated with silver nanoparticles shaped like nanospheres. In addition to these particles resembling most particles found in the literature for analogous materials, they have a preferential location on the edges and folds of the sheets of rGO, as will be discussed in the theoretical results.^27,28^ Such results indicate that, possibly, when the CTAB is added last, it will remain mostly in solution since there is a decrease in the adsorption sites available on the GO sheets since silver ions are occupying these sites. This greater distribution of silver ions increases the number of nucleation sites, which, together with the greater availability of surfactant, results in nanometer-sized particles. The CTAB can then act as a passive agent for the particles.

However, when CTAB is added before silver, according to the CTAB + 10Ag and CTAB + 30Ag materials (Figure 1d1,d2,e1,e2), the molecules protect the functional groups and cover the GO. This covering of the sheets by the CTAB has some direct effects on the characteristics of the materials: (i) preventing the metal from acting as a link between the GO sheets, reducing the structure of the material, and (ii) allowing a greater growth of the particles. Therefore, the addition of CTAB last is fundamental for the formation of particles of nanometric size, making it easier for the metal to act as a link between the sheets, making the macrostructure more compact.^27,29−31^ We performed a computational study to better understand this effect, which will be discussed in detail in the next section.

In the 30Ag + CTAB material, the concentration of the metal precursor agent is increased to 30 mM AgNO_3_, and the surfactant concentration is maintained at 1 mg mL^–1^. In Figure 1 (g1 and g2), it was found that an agglomeration of the silver particles occurs, forming larger particles compared to those of the material 10Ag + CTAB, indicating that the increase in the metal precursor results in an increase in the size of the particles.

### 3.2 CG Simulation Insights

**Figure 2:**
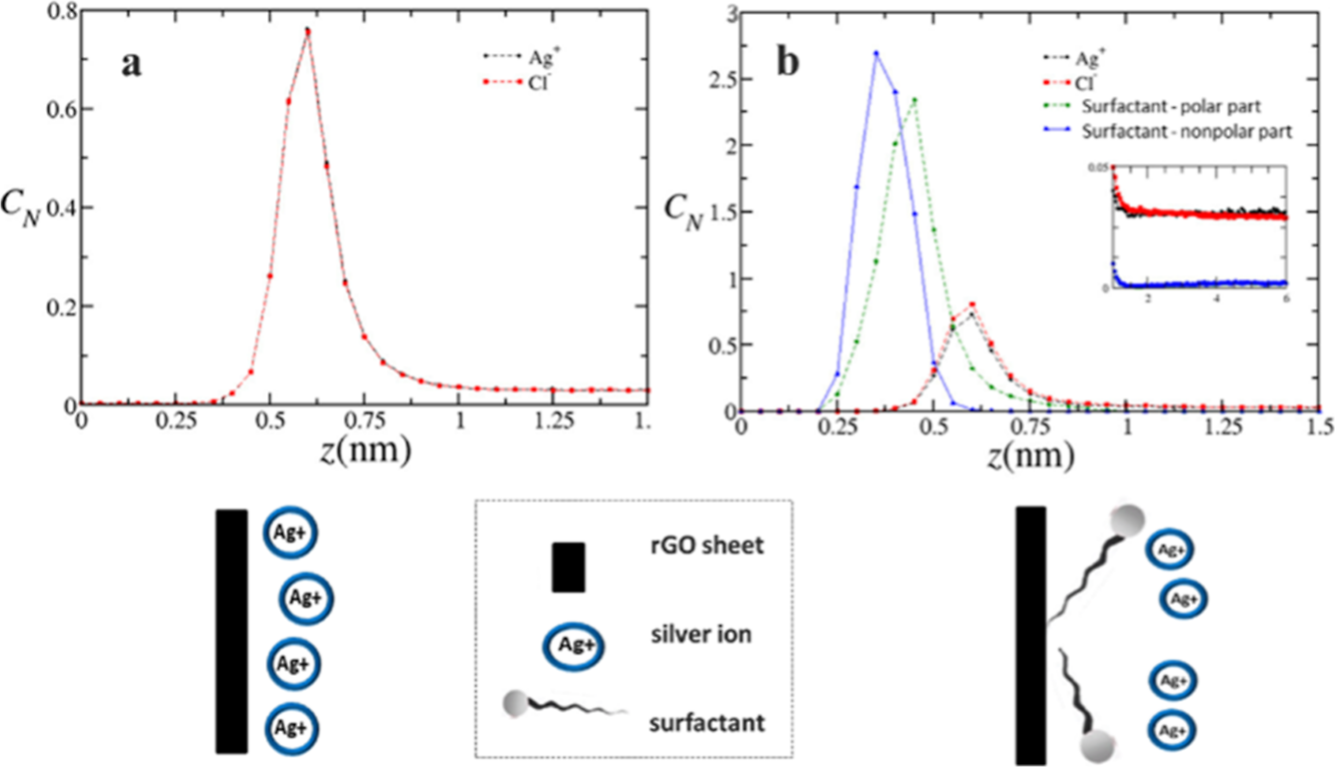
(a) Concentration profile for 10 mM silver chloride and 0 mM CTAB surfactant and (b) concentration profile for 10 mM silver chloride and 3 mM CTAB surfactant.

The theoretical results obtained in this work help to clarify the mechanisms behind the formation of the various structures observed in this experimental study. For instance, silver particles serve as connectors between rGO sheets that form the macrostructure, which also leads to the formation of silver nanoparticles. Initially, theoretical simulations were conducted to model the synthesis of nanocomposites containing silver at concentrations of 10 mM and 30 mM. These simulations were performed by adding 10 mM silver salt (specifically silver chloride) without any surfactant, along with the rGO sheet. The concentration profile of silver (CN) as a function of the distance from the rGO sheet (Z) can be seen in Figure 2a. Silver chloride was chosen because the interaction is primarily governed by charge; therefore, for this simulation, there is no significant difference between using chloride or nitrate as the counterion.

In Figure 2a, aided by the schematic, it can be observed that most silver ions are adsorbed onto the rGO sheet located at *z* = 0. The 0.5 nm distance corresponds to the excluded volume, which accounts for the size of the ions, monomers, carbon atoms, oxides, and the hydration layer of each species making up the rGO sheet. Additionally, it is evident that even at a concentration of 10 mM silver salt, the system is saturated. The silver concentration remains high, so a large portion of the ions is not utilized, causing the profile to not reach zero as the distance from the rGO sheet increases. As demonstrated in the experimental results, in the absence of CTAB, silver ions stay adsorbed onto the rGO sheets, forming silver particles that act as links between the sheets. This theoretical result illustrates the mechanism behind forming a more compact structure when the structure is decorated with silver particles.

Simulations were also performed with the addition of the 3 mM CTAB surfactant, a concentration similar to that used in the experimental study, alongside 10 mM silver chloride and the rGO sheet. The concentration profiles of silver ions and CTAB molecules can be seen in Figure 2b. In Figure 2b, it is observed that when a surfactant is added, its nonpolar tail adsorbs onto the rGO sheet, specifically in the regions where oxygenated functional groups are absent, since both the tail and the rGO sheet are nonpolar. On the other hand, the surfactant’s polar head extends further away from the sheet as it is attracted to water, which is implicitly included in the system. Silver ions are then adsorbed onto the surfactant molecules. As seen in the experimental results, in the presence of CTAB, the surfactant molecules coat the GO sheets, protecting the functional groups and preventing silver from acting as a connector between the GO sheets. This result suggests that CTAB should be added after silver so that the metal can first act as a link between the GO sheets, promoting the formation of a more compact macrostructure while leaving a greater number of CTAB molecules available for silver nanoparticle passivation.

**Figure 3:**
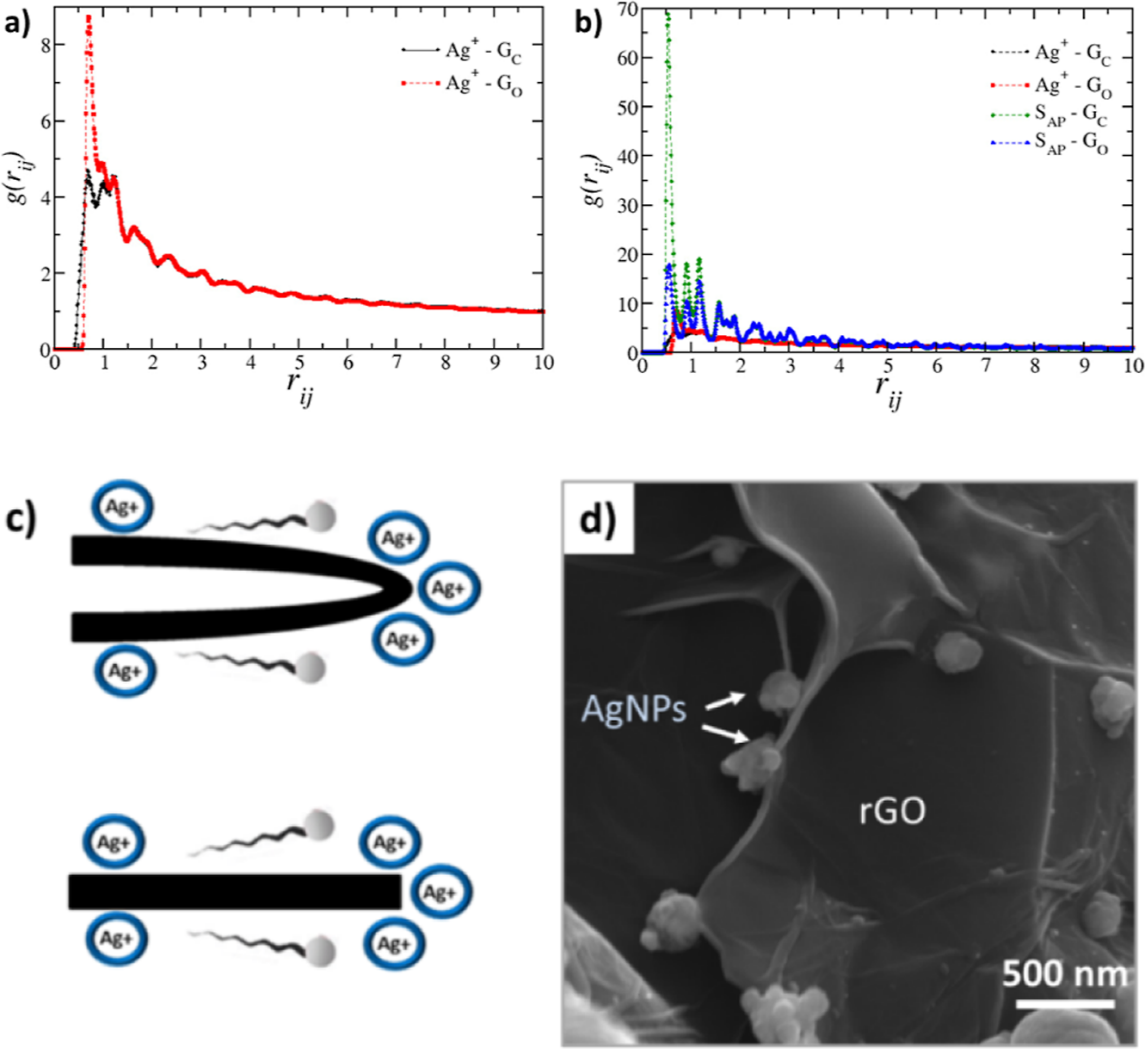
Radial distribution function (a) for 10 mM silver chloride and 0 mM CTAB surfactant and (b) for 10 mM silver chloride and 3 mM surfactant. (c) Representation of the geometric effect of the edge and fold of the rGO sheet for nucleation of silver nanoparticles and the (d) same effect observed by SEM in the sample 10Ag + CTAB (1f).

In Figure 3, g_(rij)_ represents the radial distribution function, which calculates the probability of finding a given species at a distance rij from another species. Figure 3a shows the distribution for the case of 10 mM silver chloride without a CTAB surfactant, illustrating that silver ions (Ag^+^) prefer to remain near the rGO (GO) oxide groups. In contrast, Figure 3b presents the distribution for 10 mM silver chloride with the 3 mM CTAB surfactant, showing that with the surfactant present, the nonpolar tails of the surfactant (SAP) are much more likely to approach the unoxidized regions of the rGO sheet (GC) than the silver ions.

This result supports the conclusion that if the surfactant is already present in the solution with rGO, it will not be available to passivate the silver, preventing the formation of nanometer-sized particles. The theoretical study also revealed a preferential edge effect where silver particles are more likely to form at the edges and folds of the rGO sheet (Figure 3c). In the theoretical model, silver ions accumulate at the edges regardless of the presence of polar oxygenated functional groups. In other words, the increased polarity at the edges is not the primary factor driving the accumulation of silver ions. Instead, the geometric properties of the edges and folds provide greater surface availability for the adsorption of silver ions, as shown in Figure 3b. This suggests that the edges and folds of graphene sheets are excellent nucleation sites for metallic particles, independent of the polarity of these regions. Moreover, near the edges and folds of the rGO sheet, the silver ions can still retain their hydration layer because, in the theoretical model, the rGO sheets are implicitly surrounded by water. This makes it energetically more favorable for the ions to remain in these regions, unlike the surfactant tails, which preferentially locate themselves in the nonpolar regions at the center of the rGO sheets due to their hydrophobic nature. This same behavior is observed in the experimental results obtained via SEM, as shown in Figure 3d.

The computational results presented in this study provide a molecular-level understanding that complements and validates the experimental findings, particularly regarding the role of CTAB and silver ions in shaping the morphology of the 3D rGO macrostructures. The simulations reveal that silver ions preferentially adsorb onto the edges and folds of rGO sheets, leading to the nucleation and growth of nanoparticles in these regions. This preferential edge effect, observed experimentally via SEM, is driven by the geometric availability of surface sites rather than the polarity of the functional groups. These findings elucidate the mechanism behind the formation of compact, well-decorated macrostructures in the absence of CTAB.

Moreover, the simulations clarify the critical role of CTAB in passivating silver ions and preventing their aggregation. When CTAB is added after the silver precursor, it primarily adsorbs onto the unoxidized regions of rGO, leaving silver ions free to act as connectors between the sheets. This sequence promotes the formation of smaller, more uniformly distributed silver nanoparticles, as corroborated by the experimental SEM and X-ray diffraction (XRD) results. In contrast, adding CTAB before the silver precursor leads to a reduction in the available adsorption sites for silver ions, resulting in less compact structures with larger particles. These molecular-level insights directly enhance our understanding of how the reagent addition order and concentration influence the final material properties, providing a framework for rational design in future material synthesis.

### 3.3 X-ray Diffraction

**Figure 4:**
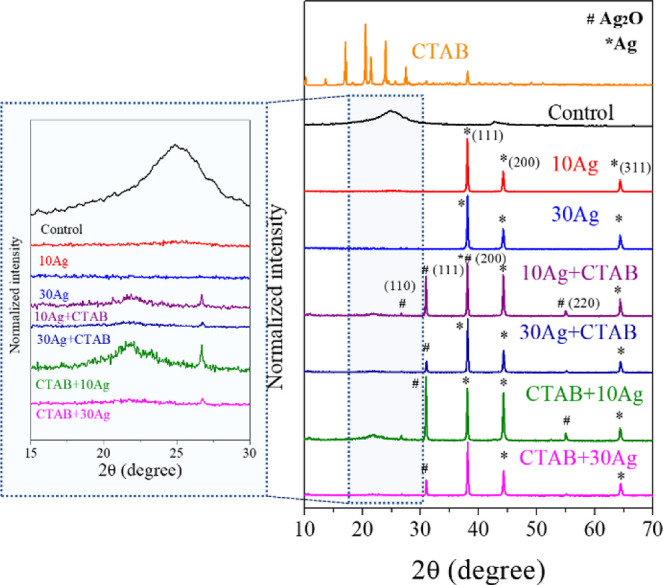
X-ray diffractograms of the samples: Control, 10Ag, 30Ag, CTAB + 10Ag, CTAB + 30Ag, CTAB + 10Ag, and pure CTAB. (*) JCPDS 87-0720 (Ag) and (#) JCPDS 76-1393.

Figure 4 presents the diffractograms of the different macrostructures obtained. For the samples decorated with silver, the X-ray diffractograms 10Ag, 30Ag, CTAB + 10Ag, CTAB + 10Ag, and CTAB + 10Ag were normalized in relation to the peak referring to the silver plane (111).

The sample control diffractogram presents two enlarged peaks with a center at 2θ = 24.9° and 42.8°, associated with a disordered stacking of rGO sheets in an arrangement different from that of the traditional graphite. This diffraction pattern is characteristic of three-dimensional materials based on graphene.^32^ These enlarged peaks can still be observed in other samples, as evidenced by the highlighted region.

Through the diffractograms of the 10Ag and 30Ag macrostructures, three peaks of strong Bragg diffractions at approximately 38.5°, 44.3°, and 64.8° corresponding to the planes (111), (200), and (311), respectively, can be indexed according to the planes of face-centered cubic silver.^33,34^ In addition, for the samples prepared in the presence of the CTAB surfactant, other diffraction peaks at 27.9°, 32.3°, 38.2°, and 55.1°, corresponding to (110), (111), (200), and (220) Ag_2_O planes, can be indexed, respectively. These peaks corroborate those of the standard Ag (JCPDS 87-0720) and Ag_2_O (JCPDS 76-1393). No plane associated with CTAB is observed in the macrostructures, indicating that CTAB does not remain in the original organization, as it is probably under the rGO sheets and stabilizing the particles.

These observations show that in the absence of CTAB, the macrostructures formed only have metallic silver particles; in the presence of CTAB, the XRD diffractograms confirmed the presence of Ag metal coexisting with Ag_2_O and that in materials prepared with a lower silver content, there is an increase in the proportion of Ag_2_O/Ag. When CTAB is added first, the proportion of oxide is greater than that of metallic silver. When a smaller amount of silver is added, as shown by the theoretical results, the CTAB molecules become more organized, forming planar micelles adsorbed on the GO. When the amounts of silver are greater, the CTAB molecules are found in greater numbers adsorbed on the silver, carrying out the passivation process of the particles. This means that samples with less silver are less passivated and, consequently, easily oxidized.

### 3.4 Thermal Properties

**Figure 5:**
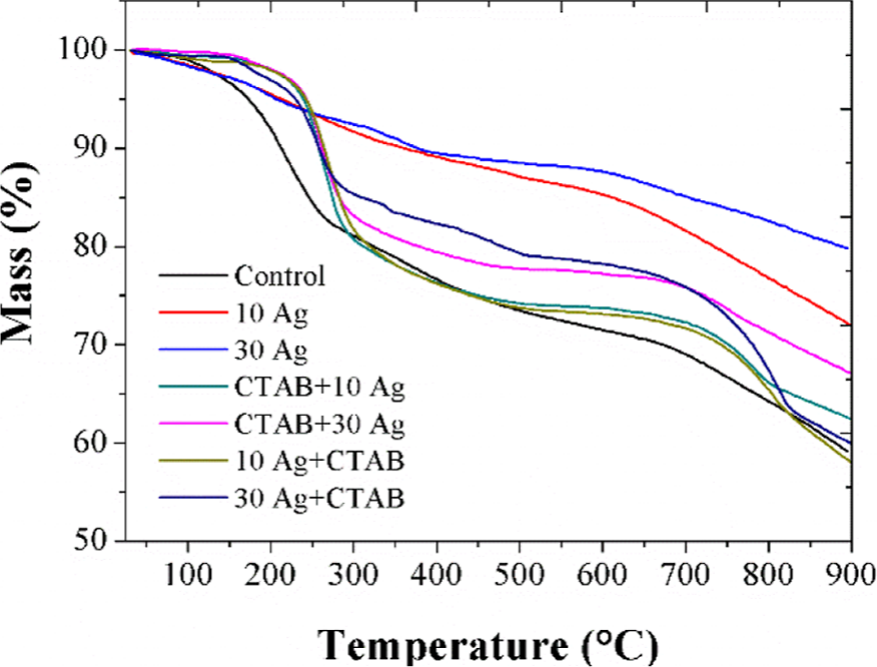
TGA curves of the different macrostructures collected in a nitrogen atmosphere.

The effects of the different synthesis methodologies on the thermal properties of the macrostructures used were evaluated by TGA under a nitrogen atmosphere. The TGA curves of the materials can be seen in Figure 5.

When analyzing the thermograms, two main mass loss events are noted for all materials. The first, from room temperature to 300 °C, is associated with water loss, elimination of oxygenated functional groups, and degradation of CTAB. For all macrostructures, a second mass loss event occurs between 600 and 800 °C, associated with the degradation of the graphitic structure through pyrolysis.

It is observed that the degradation event takes longer to start in the samples with silver to materials containing AgNPs than in the Control sample, mainly in samples 10 Ag and 30Ag, indicating that the material has greater thermal stability. It is also evident that the addition of silver favors the loss of functional groups. Once the event becomes more discrete, evident in the curves of the 10Ag and 30Ag samples, it is also noted that these two materials present the lowest mass loss and, consequently, a greater final amount of residue than other macrostructures, as expected due to the higher proportion of silver in these nanocomposites.

The addition of the CTAB surfactant decreases the thermal stability of macrostructures compared to those synthesized without CTAB. This behavior was previously described by Matos et al.,^35^ which shows that the CTAB presence negatively affects the thermal properties of nanocomposite materials. All materials with surfactants showed greater thermal stability and less mass loss than the control. However, compared to materials without surfactants, the thermal stability is lower and the mass loss is higher. It is also believed that the mass loss events around 600–800 °C observed in the samples with CTAB may be related to possible oxidation of the rGO carbon skeleton through the reduction of Ag_2_O in a N_2_ atmosphere. Additionally, given that the mass of rGO is the same in all samples, an evaluation of the residue reveals that the presence of CTAB decreases the metal content in the nanocomposite, while the order of reagent addition does not influence the amount of metal formed.

**Figure 6:**
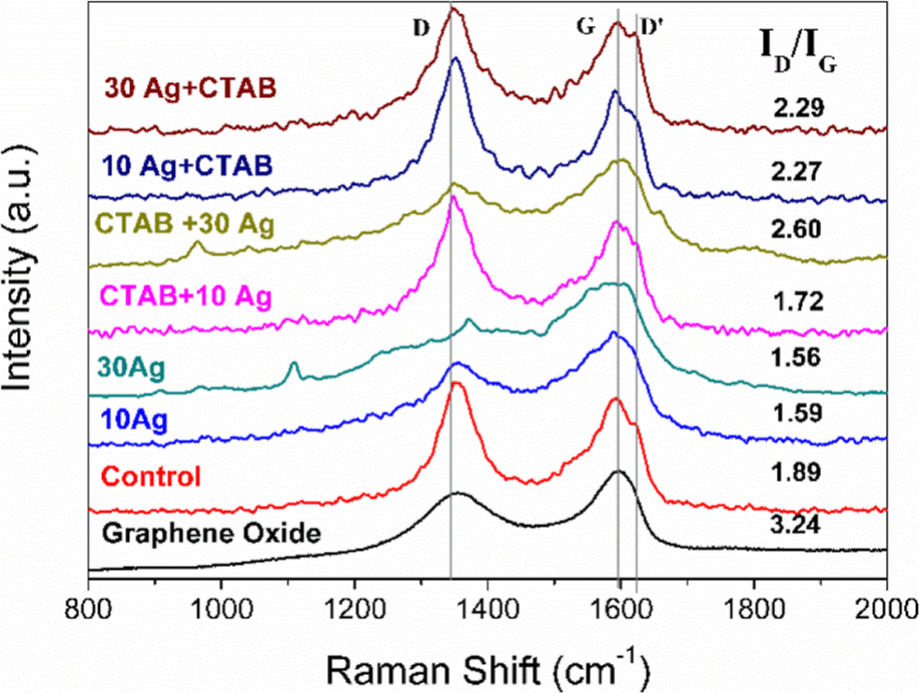
Raman spectra of neat GO, Control, 10Ag, 30Ag, CTAB + 10Ag, CTAB + 30Ag, and CTAB + 10Ag samples. Range of 800 to 200 cm^–1^.

It is worth noting that Raman spectroscopy measurements were performed for all of these samples (Figure 6). Raman spectroscopy gives carbon-based materials, somewhat like a fingerprint, that for graphene materials and their derivatives, it takes place through D bands, at approximately 1350 cm^–1^, G band at approximately 1580 cm^–1^ and, lastly, the band D′ in approximately 1610 cm^–1^.^36^ In addition, changes in these bands demonstrate the specificities of each material. Evaluating the spectrum, the structural diversity of the samples becomes evident. The *I*_D_/*I*_G_ ratio in three-dimensional graphene materials decorated with silver nanoparticles can be influenced by various factors, such as defects introduced during the decoration process, the size of the silver nanoparticles, the degree of graphene reduction, doping by the nanoparticles themselves, and intrinsic chemical and/or physical interactions. Simply reducing the GO to form a 3D rGO (control sample) led to a decrease in the *I*_D_/*I*_G_ ratio. This occurs because the reduction process removes oxygen-containing groups and restores the aromatic rings. In samples prepared without a surfactant, a reduction in this ratio was observed, possibly due to masking effects caused by nanoparticles partially covering defect sites, thereby reducing their detection in the Raman spectrum. Additionally, charge transfer or chemical bonding between graphene and nanoparticles may alter the relative intensity of the bands. In these samples and the CTAB + 30Ag sample, the appearance of additional bands was noted. These are likely related to vibrational modes activated or intensified by the direct interaction between the functional groups of graphene remnants (in the case of CTAB + 30Ag, with CTAB) and the silver nanoparticles. The interaction with silver nanoparticles and CTAB may introduce new defects and increase the *I*_D_/*I*_G_ ratio. The presence of these two species can create defects in graphene, such as vacancies, doping, or distortions in the crystal lattice, which enhance the intensity of the D band. This effect is particularly noticeable in samples containing CTAB, which exhibited significant increases in the *I*_D_/*I*_G_ ratio.

### 3.5 Electrochemical Properties and Application as a Furosemide Sensor

In this application, improvement in the electrochemical properties of the materials was evaluated according to the synthesis route used. The initial tests showed better electrochemical performance for the 30Ag sample, synthesized without the surfactant and with the highest amount of the silver precursor agent, and for the 10Ag + CTAB sample, which has the smallest particle size.

**Figure 7:**
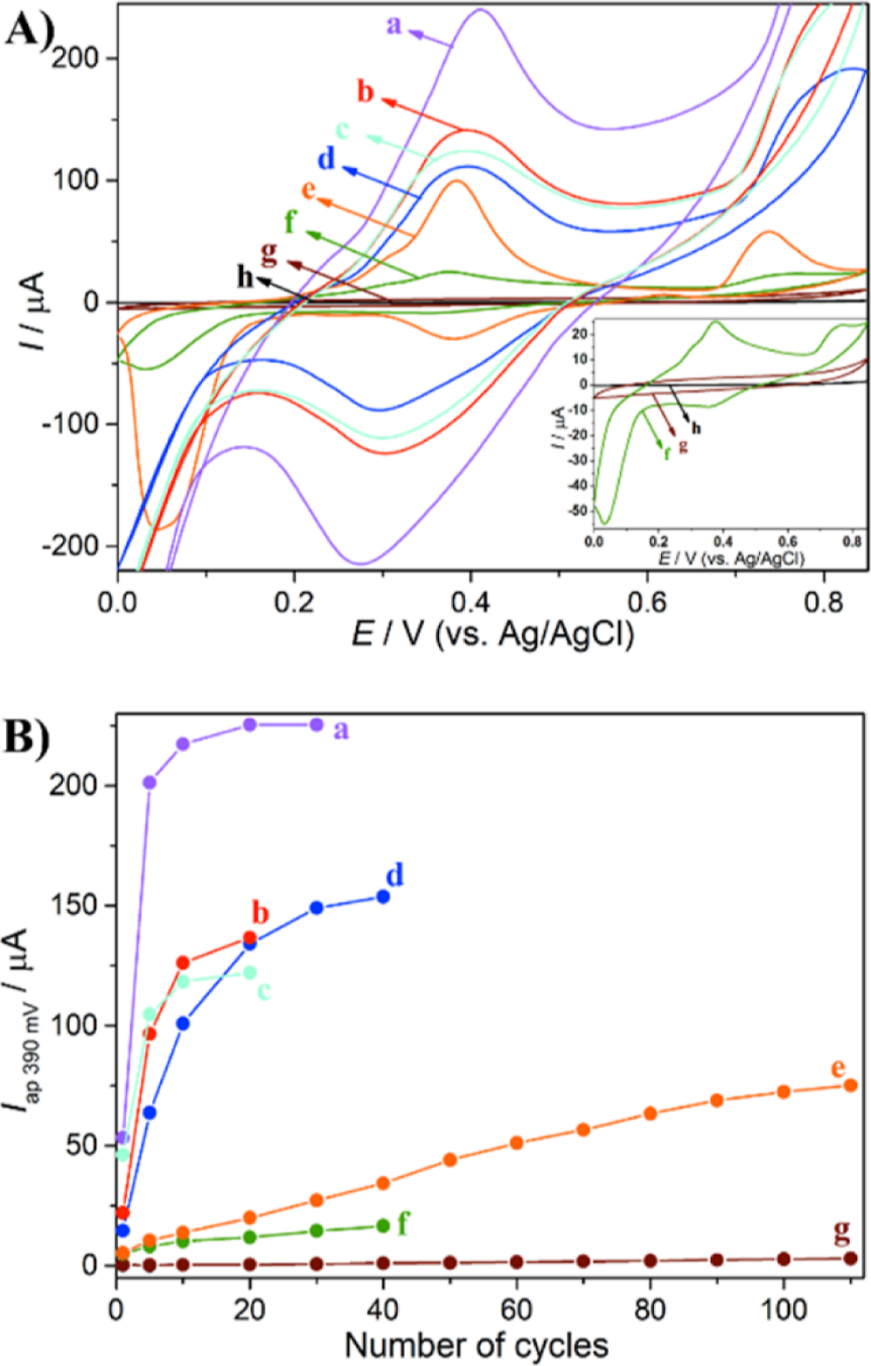
(A) Cyclic voltammograms and (B) anodic peak current at ∼0.390 V as a function of cycling of (a) 10Ag + CTAB, (b) CTAB + 30Ag, (c) 30Ag + CTAB, (d) CTAB + 10Ag, (e) 30Ag, (f) 10Ag, (g) Control, and (h) nonmodified CPE. The inset in A shows the cyclic voltammograms magnified. The cyclic voltammograms were collected in an aqueous solution of 0.1 mol L^–1^ KOH at 50 mV s^–1^.

The electrochemical behavior of the Ag-based nanocomposites was evaluated in a KOH aqueous solution, and the cyclic voltammograms are shown in Figure 7a. It is clear to see several redox processes that are related to the redox pairs Ag/Ag_2_O (or AgOH), at ∼0.4/<0.1 V, and Ag_2_O (or Ag(OH))/Ag(OH)_2_, at >0.7/<0.4 V.^37^ However, the nanocomposites exhibited different current intensities. The nanocomposites 10Ag and 30Ag presented lower current intensities, probably due to the higher particle size, leading to a lower electroactive surface area. 30Ag showed higher current intensity than 10Ag due to its higher Ag content.

The shift in the redox potentials for the CTAB-containing nanocomposites is probably related to the internal electrode resistance that directly impacts the nanocomposites with higher electroactive sites. The interaction between the Ag nanoparticles and CTAB could also contribute to this potential shift. The potential limit was set at 0.85 V, as higher potentials favored water oxidation and led to the formation of bubbles on the WE surface. The smaller particle size of the nanocomposites that contain CTAB also affected their electrochemical stability (Figure 7b). As can be seen, the CTAB-based nanocomposites quickly reached their anodic current stabilization for the peak at ∼0.4 V once their Ag nanoparticles were easily converted to oxides/hydroxides. In addition, these CTAB-containing samples already possess Ag_2_O, contributing to higher current intensities.

**Figure 8:**
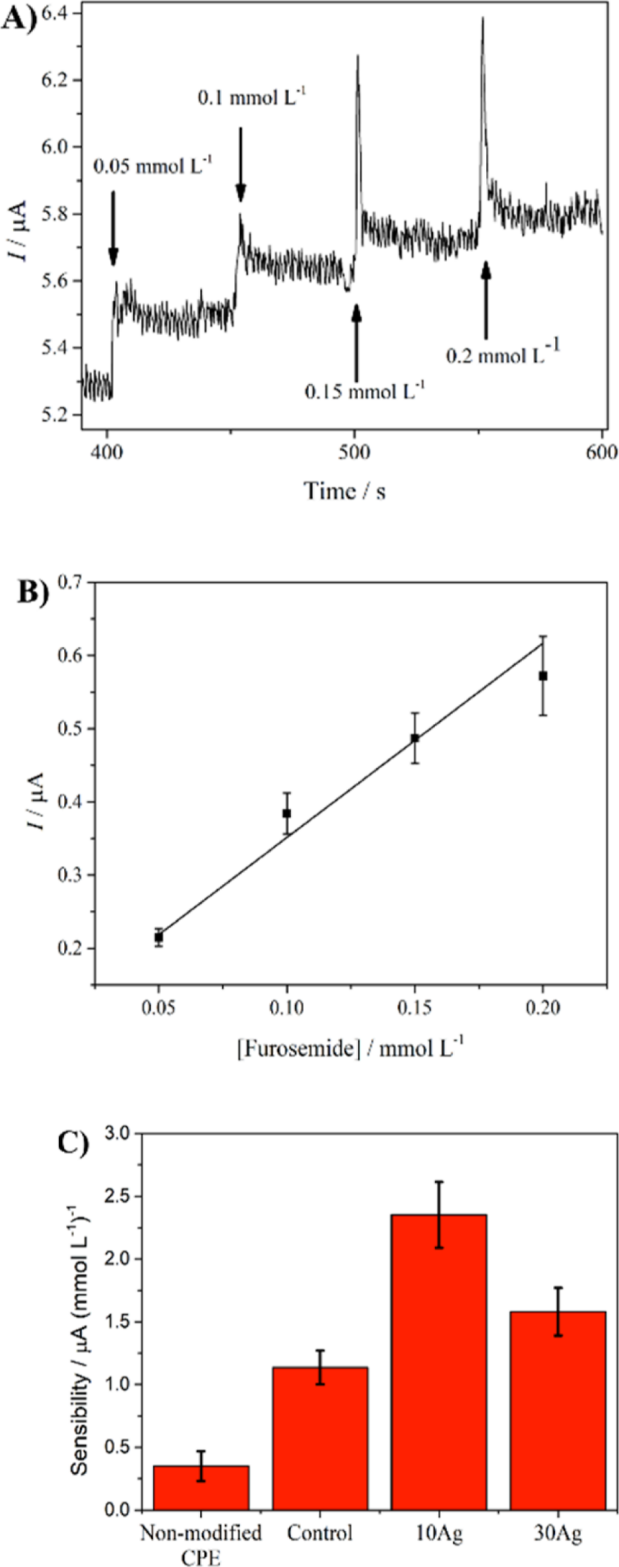
(A) Chronoamperogram and (B) analytical curve of 10 mM Ag with four additions of 0.05 mmol L^–1^ furosemide applying 0.8 V. (C) Sensitivities of the CPE modified with 10Ag, 30Ag, and control and a nonmodified CPE. The chronoamperometric analyses were obtained in a 0.1 mol L^–1^ KOH solution. The analytical curve in B was constructed from duplicated analysis and presented a correlation coefficient of 0.97.

The potential of sensing furosemide by the modified CPEs is shown in Figure 8a. This molecule has diuretic properties that make it important for the treatment of diseases such as hypertension, kidney disorder, liver disease, and fluid retention.^38^ Besides, furosemide is a doping agent commonly found in sports.^39^ The chronoamperogram of 10Ag presents current increments for the furosemide additions, indicating the analyte’s electrocatalytic oxidation by the modified CPE. However, the nanocomposites with CTAB did not present electroactivity for furosemide detection. This could be related to the surfactant covering the Ag nanoparticles (Figure S5). Figure 8b exhibits the analytical curve constructed using the current increments from the de chronoamperogram in Figure 8a. The CPE modified with 10Ag showed a sensitivity of 2.35 ± 0.26 μA (mmol L^–1^), which was higher than the 30Ag (1.58 ± 0.19 μA (mmol L^–1^)). Besides 10Ag, which possesses a lower amount of Ag than the 30Ag nanocomposite, the smaller particle size of the 10Ag nanocomposite contributed to a higher electroactive area. However, the control and the nonmodified CPE also showed significant sensitivities of 1.14 ± 0.13 and 0.35 ± 0.12 μA (mmol L^–1^), respectively. These data suggest that Ag species were not involved in furosemide oxidation through a chemical reaction. However, they increased the electroactive area of the CPE along with the carbon nanostructure.

The limits of detection (LD) and quantification (LQ) were calculated using eqs 1 and 2, respectively,^40^ where *s* is the standard deviation from the blank, and α is the sensitivity (Figure 8c). The LD and LQ for 10Ag were 21 ± 2 and 69 ± 8 μmol L^–1^, respectively. The significant values of standard deviation for both LD, LQ, and sensitivity are expected due to the heterogeneity of the Ag nanoparticle sizes in these samples. However, these low concentration values of LD and LQ indicate the potential for furosemide detection. However, this performance must be improved to reach electrochemical sensor data from the literature. For example, Khan et al. prepared polystyrene sulfonate-modified electrodes for furosemide detection in phosphate buffer using a differential pulse voltammetric technique reaching a detection limit of 2 μmol L^–1^.^41^ Dornellas et al. developed an electrochemical sensor for furosemide based on chemically reduced graphene oxide, reaching an LD of 0.7 μmol L^–1^.^42^ Brett et al. carried out the electrochemical sensing of furosemide, a composite between graphite and polyurethane, obtaining a LD of 0.15 μmol L^–1^.^38^ Other electroanalytical approaches were able to get deeper into the LD values for furosemide detection. For example, Abdel-aal et al. employed MnO_2_ nanoparticles and square wave voltammetry (SWV) for furosemide detection, reaching LD values as low as 4.44 nmol L^–1^.^43^ Nevertheless, the electrochemical characterizations described here were quite enough to understand how the morphological, structural, and composition of the nanocomposites affect their electroactivity for furosemide sensing and demonstrated their potential for sensor applications.
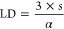
1
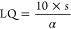
2

## 4 Conclusions

In this study, by exploring the effects of reagent concentration and addition order, we could fine-tune the characteristics of the porous macrostructures of reduced graphene oxide decorated with silver particles, including the particle size, distribution, and overall morphology. Our results show that the order of reagent addition plays a crucial role in determining the final properties of the material, with the late addition of CTAB favoring the formation of smaller silver nanoparticles that efficiently decorate the edges and folds of the rGO sheets. This finding is corroborated by both experimental observations and computational simulations, which reveal the preferential adsorption of silver ions at these sites, leading to enhanced nucleation and growth of metallic particles.

The 3D graphene network created during synthesis provided a conductive framework that maximized the available surface area for electrochemical interactions, significantly enhancing the electrochemical performance of the material. The porous structure, coupled with the unique architecture of the silver-decorated macrostructure, allowed for efficient conduction pathways, making these materials particularly well suited for electrochemical applications. It is important to note that, although promising, the proposed synthesis method requires further optimization to enhance the material’s homogeneity and achieve better control over the size and shape of the nanoparticles.

The computational results aligned well with the experimental data, providing deeper insight into the molecular-level interactions among the rGO sheets, surfactants, and silver ions. These findings highlight the importance of controlling synthesis parameters to achieve specific material properties and open new avenues for designing advanced materials tailored for various applications, particularly in electrochemical sensing.

In summary, this work presents an environmentally friendly, one-step synthesis approach for producing high-performance graphene-based macrostructures with tunable properties, showcasing their significant potential for use in a wide range of applications, including electrochemical sensors and catalysis. Since Ag is not chemically involved in the detection of the analyte but merely increases the catalytic surface area, this would facilitate the detection of other analytes. Future studies could focus on further optimizing these materials for enhanced sensitivity and selectivity in sensor applications, potentially broadening their use across diverse fields.

## Data Availability

The code developed in this study (which was used to obtain the computational results) is available at https://github.com/Bordin-Lab/espresso-scripts. All experimental data can be made available upon simple request to the corresponding author.

## Supporting Information Available

Photographic images of the freshly prepared graphene-based macrostructures, FEG-SEM images, histogram of samples, simulation setup snapshot, and chronoamperogram CTAB+30Ag with four additions of 0.1 mmol L^-1^ furosemide (PDF)
