# Supplementary material for: Structure-Dependent Properties of Silver-Decorated 3D-Reduced Graphene Oxide Nanocomposites: Influence of Reagent Addition Sequence and Applications in Electrochemical Sensing
Source: ACS Omega. 2025 Apr 17;10(16):16090–101. doi: 10.1021/acsomega.4c09382 (PMC12044435; doi:10.1021/acsomega.4c09382)
Supplement: Supplementary file 1 — ao4c09382_si_001.pdf [file ao4c09382_si_001.pdf]

Supporting Information

Structure-Dependent Properties of Silver-Decorated 3D-Reduced Graphene Oxide Nanocomposites: Influence of Reagent Addition Sequence and Applications in Electrochemical Sensing.

Paulo Castro Cardoso da Rosa[a], Anna Elisa Silva[b], Eduardo Guilherme Cividini Neiva[b], José Rafael Bordin[c], Carolina Ferreira de Matos Jauris[d]*

[a]Environmental Science and Technology Center, Federal University of Pampa, Caçapava do Sul, 96570000, Brazil.

[b]Department of Chemistry, Regional University of Blumenau, Campus 1, Blumenau, 89030-903, Brazil.

[c.]Department of Physics, Institute of Physics and Mathematics, Federal University of Pelotas, Caixa PO 354, 96001-970, Pelotas, Brazil.

[d]Department of Chemistry, Federal University of Santa Maria, Santa Maria, 97105-900, Brazil.

*CORRESPONDING AUTHOR:

Carolina Ferreira de Matos Jauris

Universidade Federal de Santa Maria

97105340– Santa Maria- RS - Brazil

Phone: +55- 55 3220-8000

E-mail: carolina.matos@ufsm.br

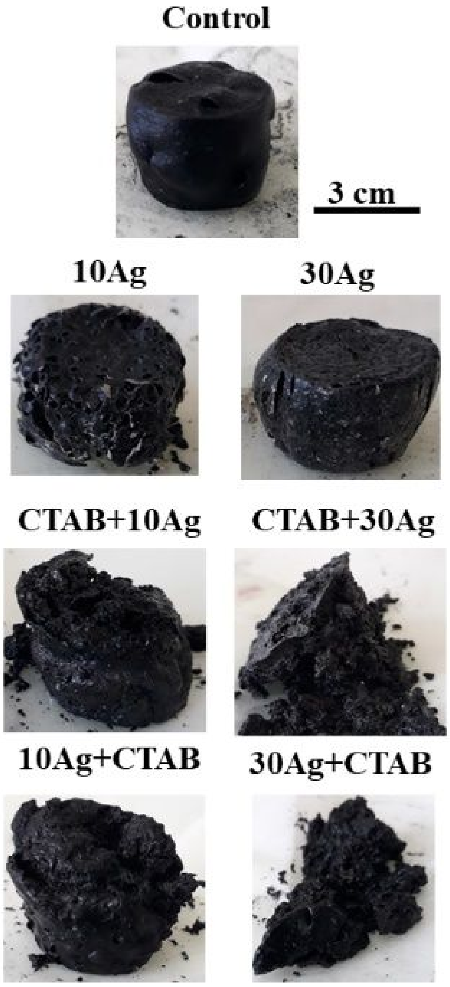

Figure. S1 Photographic images of the freshly prepared graphene-based macrostructures.

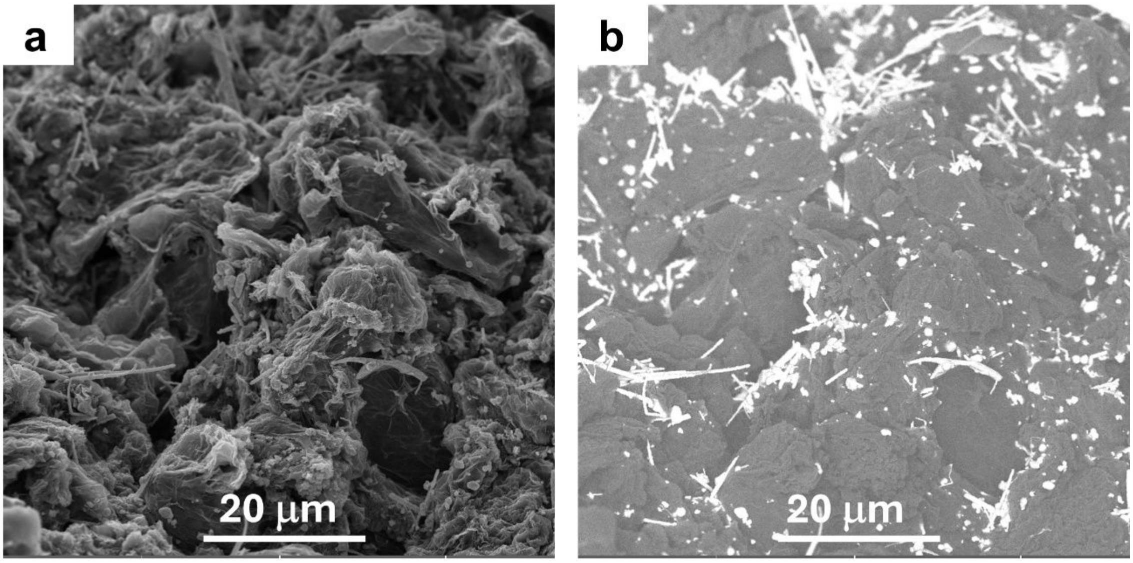

Figure S2. FEG-SEM images of a) secondary electrons and b) backscattering electrons from the same region of the CTAB+10Ag sample.

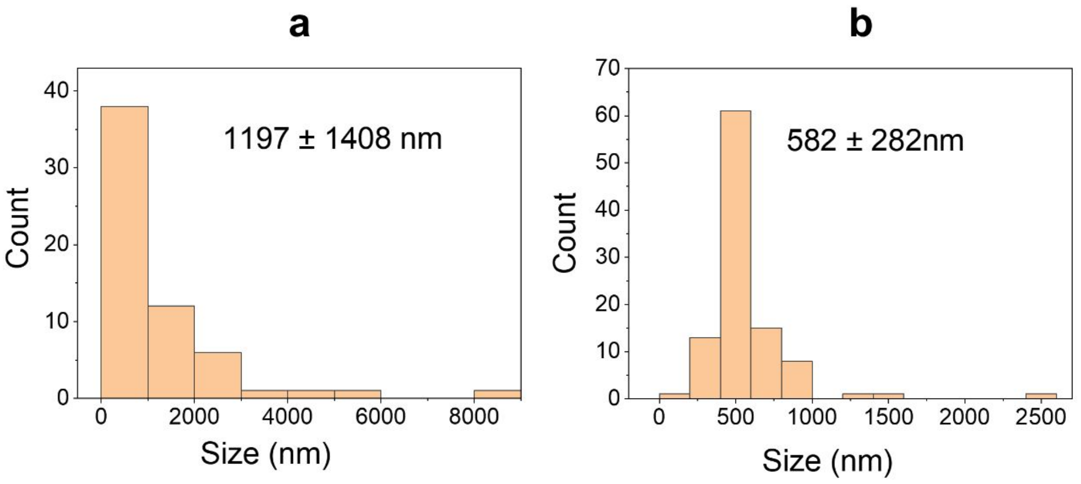

Figure S3. Histogram of samples a) 10Ag and b) 30Ag obtained from SEM images. 60 particles were counted for sample 10Ag and 101 particles for sample 30Ag.

In this work, we utilized the DRY MARTINI force field (FF)1,2 to model the system. This coarse-grained (CG) approach enabled us to simulate a large system over an extended simulation time. The CTAB parameters were obtained from Illa-Tuset et al.3. A 4-to-1 CG reduction was applied, as is standard in the DRY MARTINI FF 1,2, meaning that four atoms are represented by a single CG bead. In this framework, the hydrophobic tail of CTAB was modeled using four C1 beads, while the charged polar head was represented by a Q0 bead carrying a +e charge, following the MARTINI FF nomenclature 1,2 and with previous works for the assembly of CTAB structures in graphene sheets4. To model the oxidized graphene sheets, we adopted approaches that also employ a 4:1 mapping, where one monomer represents every four carbon atoms. These monomers, following the MARTINI force field nomenclature, were classified as C1-type beads with a 60% reduction in Lennard-Jones (LJ) parameter values4,5. Oxidized sites, when

present, were randomly selected and modeled as P5-type monomers5. For simplicity, the monomers of the oxidized graphene sheets were fixed throughout the entire integration process.

The CTAB counterions were modeled as Qa beads, while silver cations were represented by Qd beads. As usual, the intermolecular interactions were described by a combination of Lennard-Jones (LJ) and Coulomb terms. The parameters for both the LJ and Coulomb interactions are available on the MARTINI FF website: https://cgmartini.nl. The system was implemented using the ESPResSo simulation package, and an example input script has been provided as supplementary material.

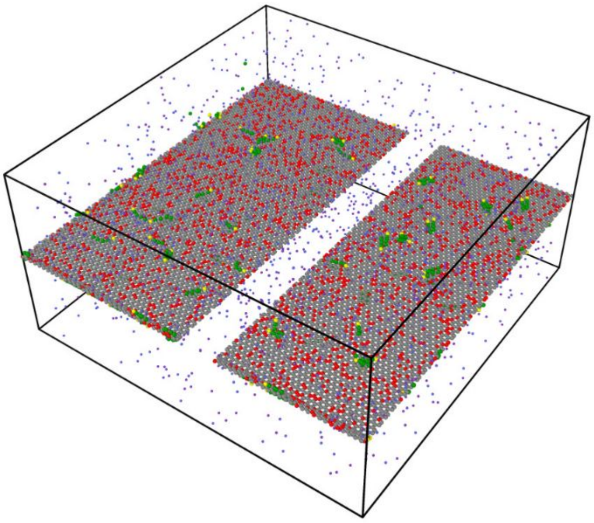

Figure S4: Simulation setup snapshot

Chronoamperogram CTAB+30Ag

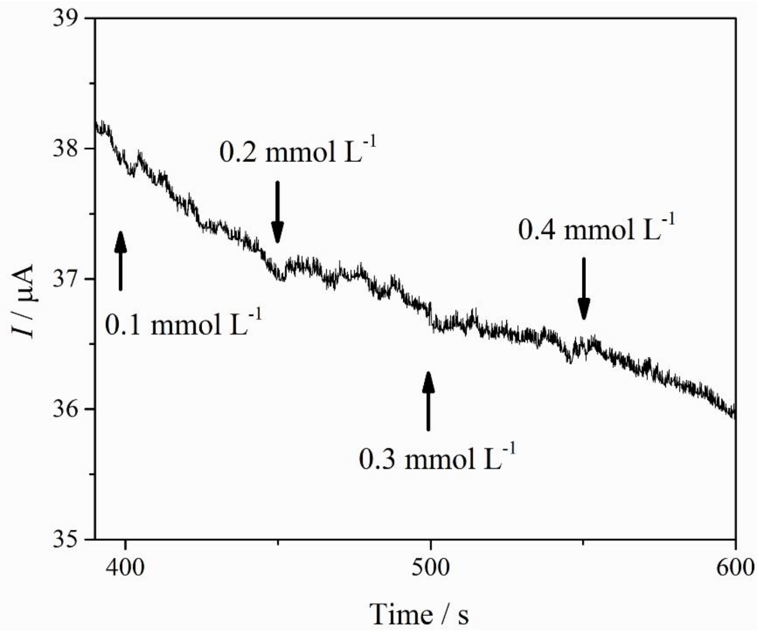

Figure S5. Chronoamperogram CTAB+30Ag with four additions of 0.1 mmol L^{-1} furosemide applying 0.8 V in an aqueous solution of 0.1 mol L^{-1} KOH.

REFERENCES

1. MARRINK, Siewert J.; TIELEMAN, D. Peter. Perspective on the Martini model. Chemical Society Reviews, v. 42, n. 16, p. 6801-6822, 2013.
2. ARNAREZ, Clément et al. Dry Martini, a coarse-grained force field for lipid membrane simulations with implicit solvent. Journal of Chemical Theory and Computation, v. 11, n. 1, p. 260-275, 2015.
3. ILLA-TUSET, Sílvia; MALASPINA, David C.; FARAUDO, Jordi. Coarse-grained molecular dynamics simulation of the interface behavior and self-assembly of CTAB cationic surfactants. Physical Chemistry Chemical Physics, v. 20, n. 41, p. 26422-26430, 2018.
4. LIU, Shuyan; WU, Dan; YANG, Xiaoning. Coarse-grained molecular simulation of self-assembly nanostructures of CTAB on nanoscale graphene. Molecular Simulation, v. 42, n. 1, p. 31-38, 2016.

5. TITOV, Alexey V.; KRÁL, Petr; PEARSON, Ryan. Sandwiched graphene-membrane superstructures. ACS Nano, v. 4, n. 1, p. 229-234, 2010.
